# A Digital Solution for an Advanced Breast Tumor Board: Pilot Application Cocreation and Implementation Study

**DOI:** 10.2196/39072

**Published:** 2023-05-18

**Authors:** Khalil Hodroj, David Pellegrin, Cindy Menard, Thomas Bachelot, Thierry Durand, Philippe Toussaint, Armelle Dufresne, Benoite Mery, Olivier Tredan, Thibaut Goulvent, Pierre Heudel

**Affiliations:** 1 Centre Leon Berard Lyon France; 2 Roche Healthcare Development Division - Roche Diagnostics France, Meylan Meylan France

**Keywords:** digital health, multidisciplinary meeting, advanced breast cancer, cancer, breast cancer, tumor, clinician, confidence, treatment, pathology, genomic, care, patient, software, data, neoplastic, pain, follow-up, electronic medical records, records

## Abstract

**Background:**

Cancer treatment is constantly evolving toward a more personalized approach based on clinical features, imaging, and genomic pathology information. To ensure the best care for patients, multidisciplinary teams (MDTs) meet regularly to review cases. Notwithstanding, the conduction of MDT meetings is challenged by medical time restrictions, the unavailability of critical MDT members, and the additional administrative work required. These issues may result in members missing information during MDT meetings and postponed treatment. To explore and facilitate improved approaches for MDT meetings in France, using advanced breast cancers (ABCs) as a model, Centre Léon Bérard (CLB) and ROCHE Diagnostics cocreated an MDT application prototype based on structured data.

**Objective:**

In this paper, we want to describe how an application prototype was implemented for ABC MDT meetings at CLB to support clinical decisions.

**Methods:**

Prior to the initiation of cocreation activities, an organizational audit of ABC MDT meetings identified the following four key phases for the MDT: the instigation, preparation, execution, and follow-up phases. For each phase, challenges and opportunities were identified that informed the new cocreation activities. The MDT application prototype became software that integrated structured data from medical files for the visualization of the neoplastic history of a patient. The digital solution was assessed via a before-and-after audit and a survey questionnaire that was administered to health care professionals involved in the MDT.

**Results:**

The ABC MDT meeting audit was carried out during 3 MDT meetings, including 70 discussions of clinical cases before and 58 such discussions after the implementation of the MDT application prototype. We identified 33 pain points related to the preparation, execution, and follow-up phases. No issues were identified related to the instigation phase. Difficulties were grouped as follows: process challenges (n=18), technological limitations (n=9), and the lack of available resources (n=6). The preparation of MDT meetings was the phase in which the most issues (n=16) were seen. A repeat audit, which was undertaken after the implementation of the MDT application, demonstrated that (1) the discussion times per case remained comparable (2 min and 22 s vs 2 min and 14 s), (2) the capture of MDT decisions improved (all cases included a therapeutic proposal), (3) there was no postponement of treatment decisions, and (4) the mean confidence of medical oncologists in decision-making increased.

**Conclusions:**

The introduction of the MDT application prototype at CLB to support the ABC MDT seemed to improve the quality of and confidence in clinical decisions. The integration of an MDT application with the local electronic medical record and the utilization of structured data conforming to international terminologies could enable a national network of MDTs to support sustained improvements to patient care.

## Introduction

Cancer care has been improved by many new therapeutic approaches in the last decade, with the emergence of immune checkpoint blockade treatment and new targeted therapies [1-4]. The wide spread of new treatments can be seen for advanced breast cancer (ABC), with the use of cyclin-dependent kinase 4 and 6 inhibitors in hormone receptor–positive ABC [5-7] and the development of many new drugs that target human epidermal growth factor receptor 2 (HER2) [8,9], resulting in an update to the classification of HER2-positive ABC [10]. These new approaches are implemented in clinical routines, and to ensure that all patients receive timely care, multidisciplinary team (MDT) meetings have been introduced in Europe, the United States of America, and most high-income countries [11-14]. Since the French law of March 4, 2002, the MDT approach in oncology has been structured with quality point requirements. MDT work is generally associated with better adherence to updated clinical guidelines [15], and to conduct such work, a detailed medical history of all presented patients should be highlighted to make the best clinical decision. However, the conduction of MDT meetings can be challenged by time restrictions, the unavailability of all members, and increased administrative work [16,17]. The development and uses of new applications have already been tested for daily health decisions [18-21].

In order to better facilitate patient case review during MDT meetings, ROCHE Diagnostics and Centre Léon Bérard (CLB) coimagined a new MDT application prototype. This digital application was tested during ABC MDT meetings, beginning in January 2021. In this paper, we discuss how this application was implemented within ABC MDT meetings at CLB and how the MDT application prototype supported clinical decisions based on accurate clinical histories.

## Methods

### Usual MDT Meeting Organization for ABC

MDT meetings are mandatory for all patients with cancer in France. ABC MDT meetings were selected as a model for evaluation and cocreation activities. The following four distinct phases were identified: (1) the instigation phase, (2) the preparation phase, (3) the execution phase, and (4) the follow-up phase.

In the first phase, a medical oncologist informs the medical assistant office that an MDT discussion is needed for a patient, who is then registered on UltraGenda (UltraGenda; instigation phase). UltraGenda is a medical appointment scheduling software used at CLB [22]. Based on time availability, the patient’s medical history is ideally prepared by medical oncologists or residents (preparation phase). The completion of this task may facilitate an MDT decision. In the execution phase of the MDT meeting, based on the UltraGenda list, each patient is discussed. Medical histories are shared by the medical oncologist in charge of the patients, and data are exposed thanks to the electronic medical record (EMR). MDT advice is audio-recorded. After the MDT meeting (follow-up phase), the medical assistant transcribes the medical advice based on the recording, and a report is added to the EMR after a final medical validation.

### Cocreation of the MDT Application

Version 1 of the MDT application was prototyped based on the challenges and needs identified during the ABC MDT audit. Subsequently, version 1 of the MDT application was used routinely for 2 months during the weekly ABC MDT meetings. After each MDT meeting, a debriefing session was held by the medical team and application development team to refine the prototype based on continuous user evaluations. After the initial 2 months, the cybersecurity for version 1 of the MDT application was evaluated before implementing version 2 of the MDT application for an ongoing routine use test.

The application was developed by an external company, in collaboration with medical oncologists of a French comprehensive cancer center.

### The MDT Application Prototype

The MDT application prototype serves as a platform that optimizes the presentation of patient cases for the purposes of MDT discussion and decision-making. The application allows for the importing and exporting of structured data based on the local EMR, imaging, and genomic pathology information. The data within the MDT application conforms to international terminologies.

Two factors—authentication and a personal Répertoire Partagé des Professionnels de Santé (RPPS) number (shared directory of health care professionals)—are needed to access MDT application.

### Practical Methods of the Audit and User Feedback Assessment

An audit was carried out prospectively before and after the implementation of version 2 of the digital solution. The cases discussed before and after using the application prototype consisted of ABC cases only and had the same complexity level. The items assessed precisely were the total duration of an MDT meeting, the estimated time lost searching for information in the EMR, the average discussion time per patient, the percentage of clinical cases that were postponed due to a lack of information, the percentage of clinical cases that were registered but already discussed previously, the percentage of clinical cases that were registered but postponed due to a lack of time, the percentage of files processed, and the number of clinical cases that were discussed but not recorded. Based on this audit, a detailed assessment was carried out in order to determine in which phases of the process pain points were identified (the instigation, preparation, execution, or post–tumor board phase) and determine the types of pain points (those linked to problems related to the organization of the process, those linked to the technological limits of the tools used, or those linked to a lack of human resources). Similarly, a before-and-after survey was used to assess the user experience among the health care professionals involved in the ABC MDT. A questionnaire was sent to 15 health care professionals involved in the ABC MDT. The items assessed precisely were the level of satisfaction, the level of confidence in decision-making, and an open question on what would have driven any level change.

### Ethics Approval

This study was approved in October 2019 by the local data protection officer, on behalf of French regulatory authorities (Commission Nationale de l’Informatique et des Libertés), in accordance with the MR004 methodology (reference number: H001 – 002). This study adhered to the European laws for the protection of personal data (General Data Protection Regulation). All patients were informed of the possibility of their health data being used for research purposes, and none expressed an opposition to this possibility.

The implementation of the MDT application in the ABC MDT meetings did not result in changes to the rules for the application’s use; at least three different medical specialists are required to discuss each case and share the conclusions of the MDT, and a personal RPPS number must be used to access the application. The MDT application was implemented in accordance with current regulations.

## Results

### The Pain Points and Needs Identified for the ABC MDT

An audit was carried out prospectively before the implementation of version 2 of the digital solution during 3 MDT meetings, including 70 clinical case discussions. The first audit of the original ABC MDT approach identified 33 discrete pain points related to the preparation (n=16), execution (n=11), and post–tumor board (n=6) phases. No issues were identified related to the instigation phase; however, for the other three phases (the preparation, execution, and post-MDT meeting phases), multiple difficulties were identified and subsequently classified as process, technology, or resource issues. In the preparation phase, 8 difficulties were identified with processes (eg, the lack of a systematic approach to informing the medical question and the overbooking of cases with a lack of transparency on time available), 5 pain points were related to technology, and 3 pain points concerned resources. In the execution phase, 6 pain points were related to processes, 3 were related to technology, and 2 were related to resources. In the follow-up phase, there were 4 pain points related to processes, 1 was related to technology, and 1 was related to resources (Table 1).

**Table 1:** Pain point distribution by tumor board phase.

Pain point type	Instigation phase	Preparation phase	Execution phase	Follow-up phase	Total, N
Process, n	0	8	6	4	18
Resources, n	0	3	2	1	6
Technology, n	0	5	3	1	9
All pain points, N	0	16	11	6	33

### New Approach for Tumor Boards Involving a Cocreated MDT Application

#### Instigation Phase

No issues were identified for this phase. The process for this phase remained the same; a medical doctor informs the medical assistant office of the need for a patient to be registered on UltraGenda for discussion at an upcoming MDT meeting.

#### Preparation Phase

In the preparation phase, a nurse navigator uses structured data from the MDT application to systematically prepare a patient case.

#### Execution Phase

In the execution phase, patients are discussed by the MDT based on lists generated by UltraGenda. The MDT application presents a single-slide timeline visualization of the medical history, patient characteristics, and previous treatments, superseding the use of the EMR for case presentation. The documentation of case decisions is now captured, structured, and validated within the MDT application, through which an autogenerated MDT report is created as a permanent record. This replaces the use of audio-recorded case decisions.

#### Post–Tumor Board Phase

**Figure 1:**
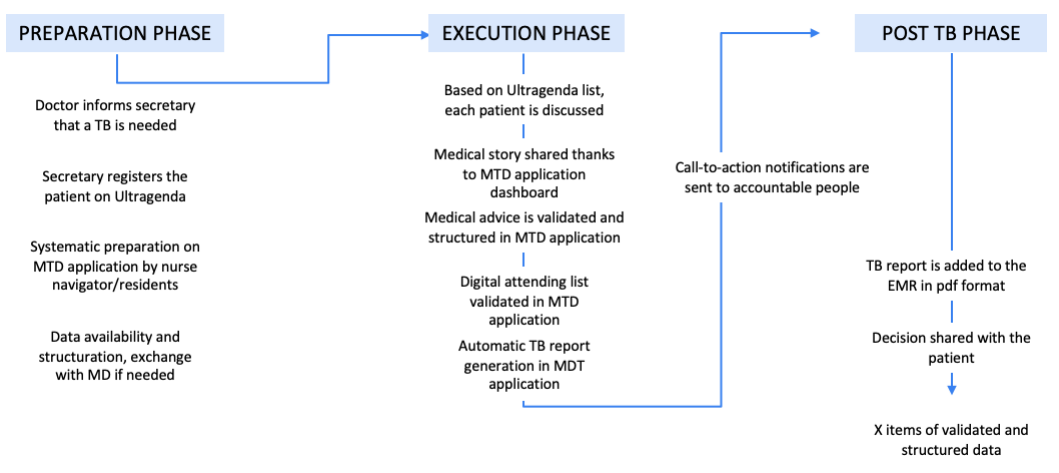
Diagram of the new process for MDT meeting organization via the MDT application. EMR: electronic medical record; MD: medical doctor; MDT: multidisciplinary team; TB: tumor board.

An autogenerated MDT report in PDF format is added to the EMR at the conclusion of the meetings. Subsequently, call-to-action notifications are sent to accountable individuals. Patient data remain in the MDT application in a structured format for potential use in future meetings and for audit and reporting purposes. An overview of the new process is shown in Figure 1.

### Improvements Made by the ABC MDT After the Implementation of the New Process Involving the MDT Application

A second audit was carried out prospectively after the implementation of version 2 of the digital solution during 3 MDT meetings, including 58 clinical cases.

After the implementation of the MTD application, the time dedicated to patient case discussion slightly decreased, and the percentage of cases for which a therapeutic recommendation was made improved. The mean discussion times per patient were comparable (legacy approach: 2 min and 22 s; new approach: 2 min and 14 s). The total time per meeting dedicated to case discussions fell from 53 minutes and 20 seconds to 42 minutes and 40 seconds; however, this was predominantly driven by the lower average case numbers per meeting (22.7 cases vs 16.3 cases). Most interestingly, no case postponements occurred after the introduction of the MDT application, whereas the legacy process had an average case postponement rate of 31%.

### User Feedback Assessments

A before-and-after survey was used to assess user experience. The web-based questionnaire was sent to 15 health care professionals involved in the ABC MDT meetings, and of these 15, 8 (53%) responded. After the introduction of the MDT application, the mean level of satisfaction (a score out of 5) improved from 3.4 to 4. In addition, the mean confidence in decision-making (a score out of 10) improved from 5.6 to 8. The main drivers for this were the standardized presentation of cases and patient history preparation by an oncology nurse navigator or by oncology residents.

## Discussion

Optimal decisions for patients with cancer have been related to MDT care [11]. Since its implementation as a regular practice, MDT meetings have shown an impact on management plans, patients, and process outcomes [14]. Nevertheless, successful MDTs require time and coordination for a specialist group of health care professionals to meet regularly, as well as additional time to prepare cases [15]. Considering the increasing number of patients and the increasing complexity of the clinical cases discussed, it appears that the average discussion time for a clinical case is around 5 minutes [23]. This proves the need for intelligent computing systems that integrate and analyze clinical data from the EMR to enable better clinical decision-making.

In CLB, the ABC MDT conducted an audit to optimize its functioning. This assessment identified 33 pains points that were used to inform the development of a new process for ABC MDT work. Difficulties concerned the process (18/33, 55%), the technology (9/33, 27%), and the lack of available resources (6/33, 18%; Table 1). Based on these observations, ROCHE Diagnostics and CLB imagined a new process for the ABC MDT meetings that would be enabled by a dedicated MDT digital application. The introduction of the MDT application into the MDT meetings improved the likelihood of reaching a decision, as this resulted in discussions only for cases where all the required information was available. Moreover, user feedback showed that participants had increased confidence in the decisions made. It is likely that this was due to the improved presentation of data on the MDT application dashboard, as it displays a single-slide timeline visualization of previous treatments, tumors, and patient characteristics, and its use replaces the time-consuming and frustrating process of searching for various key information within distributed reports that the EMR may or may not contain.

The main change enabled by the new process was the systematic preparation of patient medical histories by the oncology nurse navigator or by oncology residents in a structured format within the MDT application. Further, the automatic generation of the MDT decisions removed the need for audio recordings of decisions and additional work to manually record the conclusions.

The main limitation of our results is that the MDT application has only been tested in ABC MDT meetings, limiting its implementation for localized breast cancer boards or other metastatic histology boards. Digital solutions for MDT meetings have already been shown to significantly reduce the overall case preparation time [24]. Moreover, our study shows that an MDT application has the potential to improve MDTs’ confidence in making the best decisions for patients. Further work is needed to assess whether the use of an MDT application improves the implementation of decisions and results in better clinical outcomes.

A benefit of an MDT application that collects structured clinical data and conforms to internationally accepted terminologies is its ability to generate a real-world data set, which could be used to answer additional research questions in the future [25]. Digital tools, such as ConSoRe (Continuum Soins Recherche) [26], have been developed to facilitate the collection of large amounts of data, but these tools are limited by the heterogeneity of medical reports. It is envisioned that over time, an MDT application could serve a national network for rare tumors, such as the one supported by the French National Cancer Institute (Institut National du Cancer). This network provides diagnostic expertise and aims to improve the care of patients with rare tumors by using referral MDT boards. It can also facilitate recruitment for clinical trials that are dedicated to only rare cancers and involve international efforts.

MDT meetings are important elements in the management of patients with cancer. However, the number and complexity of the clinical cases treated make organizational and technological development necessary for being able to meet medical and administrative needs. A precise evaluation of ABC MDT practices allowed for the coconstruction of an MDT application that improved the confidence of clinicians in their decisions while structuring health data.

## Abbreviations

ABC: advanced breast cancer
CLB: Centre Léon Bérard
ConSoRe: Continuum Soins Recherche
EMR: electronic medical record
HER2: human epidermal growth factor receptor 2
MDT: multidisciplinary team
RPPS: Répertoire Partagé des Professionnels de Santé

## Data Availability

The data sets generated and analyzed during this study are available from the corresponding author on reasonable request.
